# Regulatory mechanisms of circular RNAs during human mesenchymal stem cell osteogenic differentiation

**DOI:** 10.7150/thno.89066

**Published:** 2024-01-01

**Authors:** Chiara Mazziotta, Giada Badiale, Christian Felice Cervellera, Mauro Tognon, Fernanda Martini, John Charles Rotondo

**Affiliations:** 1 Department of Medical Sciences, University of Ferrara, 44121 Ferrara, Italy.; 2 Center for Studies on Gender Medicine - Department of Medical Sciences, University of Ferrara. 64/b, Fossato di Mortara Street. Ferrara, Italy.; 3 Laboratory for Technologies of Advanced Therapies (LTTA), University of Ferrara, 44121 Ferrara, Italy.

**Keywords:** circular RNA, circRNA, microRNA, miRNA, crosstalk, osteogenic differentiation, osteogenesis, mesenchymal stem cell

## Abstract

Human osteogenic differentiation is a complex and well-orchestrated process which involves a plethora of molecular players and cellular processes. A growing number of studies have underlined that circular RNAs (circRNAs) play an important regulatory role during human osteogenic differentiation. CircRNAs are single-stranded, covalently closed non-coding RNA molecules that are acquiring increased attention as epigenetic regulators of gene expression. Given their intrinsic high conformational stability, abundance, and specificity, circRNAs can undertake various biological activities in order to regulate multiple cellular processes, including osteogenic differentiation. The most recent evidence indicates that circRNAs control human osteogenesis by preventing the inhibitory activity of miRNAs on their downstream target genes, using a competitive endogenous RNA mechanism. The aim of this review is to draw attention to the currently known regulatory mechanisms of circRNAs during human osteogenic differentiation. Specifically, we provide an understanding of recent advances in research conducted on various human mesenchymal stem cell types that underlined the importance of circRNAs in regulating osteogenesis. A comprehensive understanding of the underlying regulatory mechanisms of circRNA in osteogenesis will improve knowledge on the molecular processes of bone growth, resulting in the potential development of novel preclinical and clinical studies and the discovery of novel diagnostic and therapeutic tools for bone disorders.

## 1. Introduction

Non-coding RNAs (ncRNAs) encompass a class of RNA molecules that control multiple cellular processes and functions 1. NcRNAs are primarily encoded by genes without protein-coding capability as they do not undergo the canonical post-transcriptional mRNA modification/translational processes 2-4. These molecules are classified into long and small ncRNAs (lncRNAs/sncRNAs) 5-7. The first group has recently emerged as a regulator of numerous pathways and cell processes 1,8-10. The second includes microRNAs (miRNAs), which are well-known post-transcriptional gene expression regulators 11-14. The biological function of lncRNAs and miRNAs relies on a complex regulative network in which these two molecules mutually and/or independently interact to control the expression of downstream target genes 1.

Circular RNAs (circRNAs) are a class of closed endogenous lncRNAs 15-18. The typical closed loop-structure allows a sufficient structural stability to withstand enzymatic RNA degradation 19 and confers higher stability on circRNA compared to miRNAs and linear lncRNAs 14,20. The first circRNA molecule was discovered by Sanger in 1976 21. However, molecular technology advances in the recent decades have allowed the identification of a limited number of circRNAs whose fundamental functions have remained unclear until lately 21-23. With the recent development of novel experimental approaches 24-27, such as sequencing technologies, bioinformatic tools 28 and functional experimental designs, numerous circRNAs have been identified and categorized according to function. Understanding the role of circRNAs in regulating numerous cellular processes is a continuously ongoing research area 29.

CircRNAs regulate the expression of genes predominantly through an RNA-RNA interaction mechanism. They can negatively modulate the activity of miRNAs as competitive endogenous RNAs, counteracting, in turn, the inhibitory activity of miRNAs on their mRNA targets 30. Given this regulatory activity on gene expression, it is not surprising that circRNAs, as other epigenetic players 31-34, control numerous processes, including cell metabolism, differentiation, proliferation, apoptosis and development 16,35,36. Consistently, dysregulations in the circRNA activities are related to the onset and development of various pathologies 29,37,38.

A growing number of studies have underlined that circRNAs can fulfill their regulative role on the self-renewal, proliferation and osteogenic differentiation of human mesenchymal stem cells (hMSCs) 35,39,40. hMSCs are well-known pluripotent stem cells that can be isolated from different sources, such as bone marrow, adipose tissue, and dental pulp. hMSCs can differentiate into distinct cell types, including osteoblasts, adipocytes, and chondrocytes. As such, hMSCs represent a promising biomedical solution for the treatment of bone disorders as well as osteoarticular post-operative conditions. hMSCs also exhibit important biological properties, playing a key role in both natural bone healing, as well as in bone tissue engineering approaches 1,41. In recent years, genetic and epigenetic regulation of hMSC osteogenic differentiation has accurately been studied and an increasing number of circRNAs involved in this process were identified.

The aim of this review is to draw attention to currently known regulatory mechanisms of circular RNAs during the osteogenic differentiation of hMSCs. Here, we present, describe, and discuss the most recent results obtained both *in vitro* and *in vivo* regarding the relationship between circRNAs, miRNAs and downstream osteogenic target genes.

## 2. Circular RNA biogenesis

**Figure 1:**
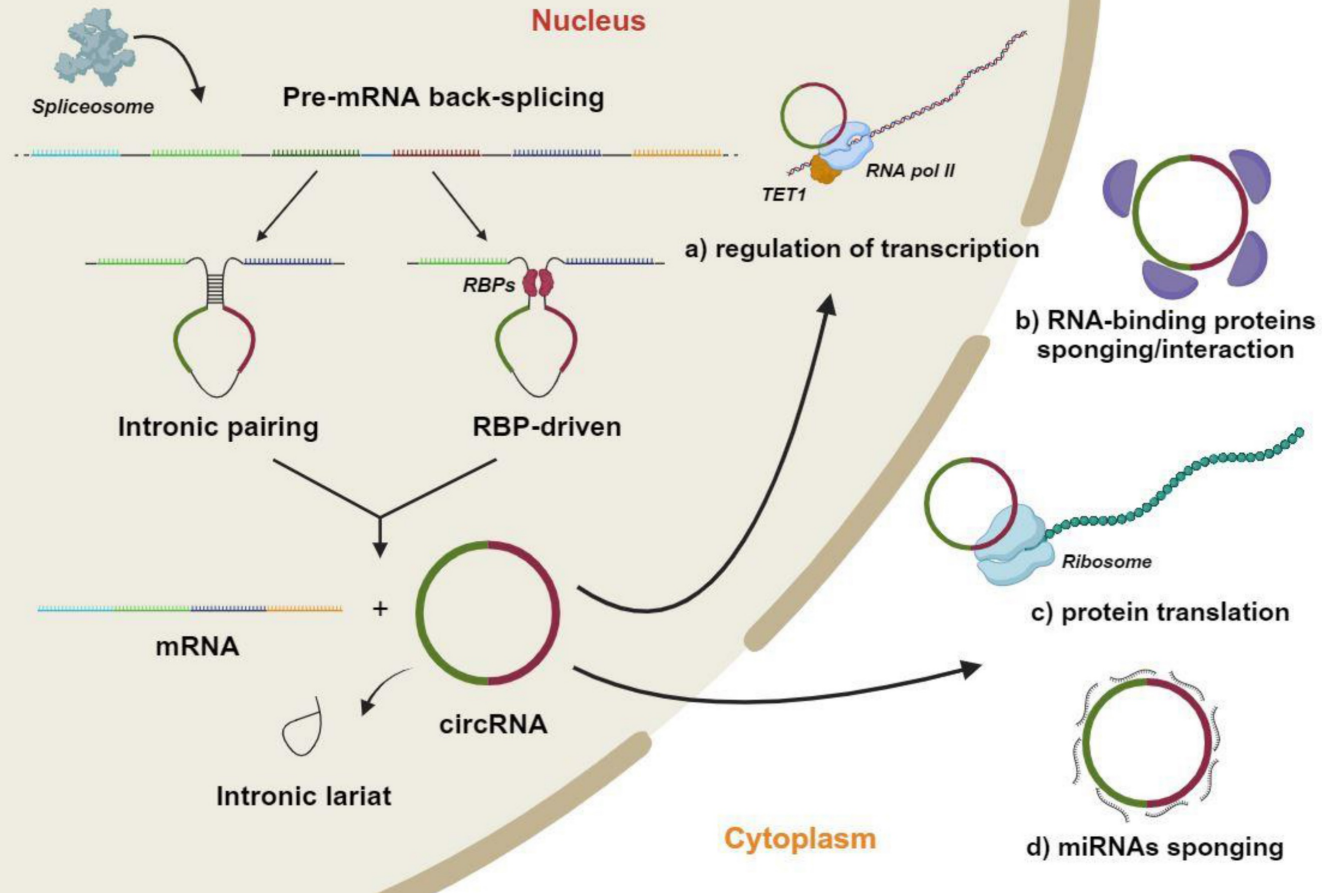
** Circular RNAs (circRNAs) biogenesis mechanisms and functions.** In the nucleus, a single molecule of pre-mRNA is subject to back-splicing by the canonical spliceosomal machinery. The generation of mature circRNA molecules, which generally contains two or three exons occurs through two different modalities. The first exploits the base complementarity between two circRNA flanked introns to create a secondary structure that makes possible back-splicing. In the second, specific RNA-binding proteins recognize/bind to specific regions of circRNAs' flanked introns thus leading to the back-splicing secondary structure formation. The final products of this process are various mature RNA molecules, including a mRNA, a circRNA and one or more intronic lariats, obtained from the removal of introns interposed between circRNA exons. Mature circRNAs are crucial molecules which regulate several cell mechanisms, such as: a) Regulation of transcription by enhancing of RNA polymerase II (RNA pol II) and Ten-eleven translocation protein (TET) promoter demethylation activities. b) Sponging and regulation of RNA-binding proteins half-life favoring proteasome mediated degradation. c) Translation of particular circRNA regions through a CAP-independent mechanism. b) Sponging-mediated negative modulation of microRNAs (miRNA). This figure was made by using the BioRender online tool (www.biorender.com).

CircRNAs are abundant endogenous RNA molecules that, similarly to other ncRNAs, originate in the nucleus 42-44 (Figure 1). However, they present a distinctive characteristic which is that the 5' and 3' ends are covalently linked by back-splicing of exons from a single pre-mRNA 15-18,45. CircRNAs therefore lack in the 5' cap and the 3' polyadenylation tail, which are two fundamental features of miRNAs 46. CircRNAs are produced by the end-to-end joining of RNA transcription fragments as a result of non-canonical splicing mechanisms being activated 47,48. The generation of a mature circRNA molecule, which contains two or three exons, provides two distinct modalities. The first exploits the base complementarity between two circRNA flanked introns, in order to generate a secondary structure allowing back-splicing to occur. In the second, RNA-binding proteins recognize and bind to specific regions of the circRNAs' flanked introns, thereby leading to the formation of the back-splicing secondary structure. Following nuclear processing, circRNAs are transferred into the cell cytoplasm 49,50. As mature molecules, circRNAs are averagely ~550 nucleotides in length with a half-life of ~48 h 49,51,52.

## 3. Circular RNA functions

CircRNAs play important functions in numerous cellular processes as key epigenetic regulators of gene expression. This activity encompasses both transcriptional and post-transcriptional mechanisms (Figure 1) 53. So called miRNA sponge activity is the most commonly described circRNA mechanism of action. This mechanism supports the endogenous RNA hypothesis, whereby specific RNA transcripts, such as circRNAs, can affect the abundance of other RNA molecules by limiting the availability of a common miRNA 54. In other words, circRNAs inhibit miRNAs to positively regulate the expression of miRNA target genes 16. Additional circRNA activities have been reported, although being limitingly investigated 55. Emerging evidence indicate that circRNAs can bind to proteins, including RNA-binding proteins, in order to regulate the expression of target genes 56,57. This specific protein-binding ability on RNA-binding proteins confers to circRNAs the ability of controlling the maturation/splicing of mRNAs 58,59. Indeed, circRNAs can affect the spliceosome activity by competing with pre-mRNA molecules, modulating, in turn, the expression of transcribed genes 56. CircRNAs can also directly mediate the sequestration of various RNA-binding proteins through a protein sponge mechanism, and further negatively regulate the expression of downstream genes. Contrariwise, several circRNAs can recruit associated proteins to specific gene regulatory locations in order to positively regulate the expression of downstream genes 60,61. Lastly, circRNAs have also been reported to act as scaffolds in the assembly of protein complexes and sequesters of proteins from native subcellular localization 62.

## 4. Regulatory activities of circular RNAs during human mesenchymal stem cell osteogenesis

### 4.1 Wnt/β-catenin pathway

**Figure 2:**
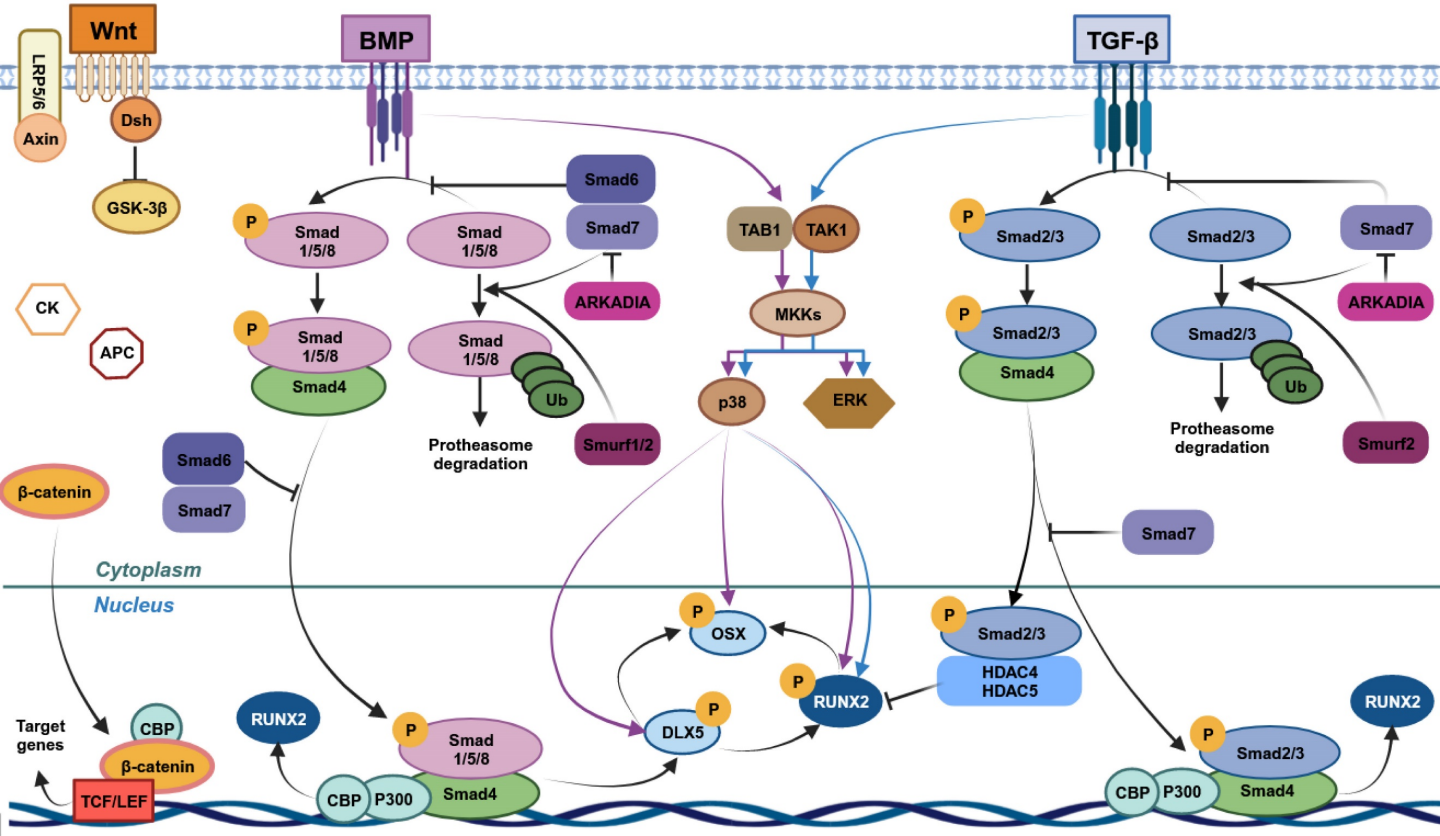
** Pathways involved in human mesenchymal stem cells (MSCs) osteogenic differentiation.** Wnt/β-catenin, bone morphogenetic protein (BMP) and transforming growth factor-beta (TGF-β) cascades are the main signaling pathways leading to MSCs osteogenic differentiation. Wnt/β-catenin induces MSCs osteogenic differentiation through β-catenin translocation into the nucleus, leading to the expression of target genes, including RUNX2. TGF-β and BMP through the binding with their respective receptors lead to the activation of Smad-dependent and -independent cascades. In TGF-β Smad-dependent signaling, Smad2/3 is phosphorylated upon ligand-receptor binding and interacts with Smad4, leading to its migration into nucleus. Here, this complex induces RUNX2 expression interacting with CBP and P300 co-activators. Smad2/3 without Smad4 interaction, forms a complex with HDAC4/5, blocking RUNX2 expression. Unphosphorylated Smad2/3 is degraded by ubiquitination. In BMP Smad-dependent pathway, BMPs receptors of type I and II (BMPR-I and BMPR-II) are activated by their ligands and lead to Smad1/5/8 phosphorylation. These molecules form a complex with Smad4 and move into the nucleus, acting as transcriptional regulator of target genes, including RUNX2 and Osterix (OSX). Unphosphorylated Smad1/5/8 is degraded by ubiquitination. This Smad-dependent cascade also comprises Smad6/7 and Smurf1/2, which are negative regulators of this pathway. In Smad-independent cascade, TGF-β/BMP signaling pathways promote the maturation and proliferation of osteoblasts inducing DLX5, RUNX2 and OSX phosphorylation through a cascade that comprises TAK1-TAB complex, ERK and p38. This figure was made by using the BioRender online tool (www.biorender.com).

Wnt/β-catenin is one of the most important pathways governing bone homeostasis 63 (Figure 2). It is primarily implicated in hMSC proliferation, self-renewal and osteogenic differentiation 64-66. Wnt includes a family of 19 glycoproteins 67, while β-catenin is a protein with a double function given its contribution to cellular adhesion and gene transcription 68. Wnt/β-catenin-driven MSC osteogenesis results in the expression of downstream pro-osteogenic genes, ultimately leading to bone formation 69*.*

**Figure 3:**
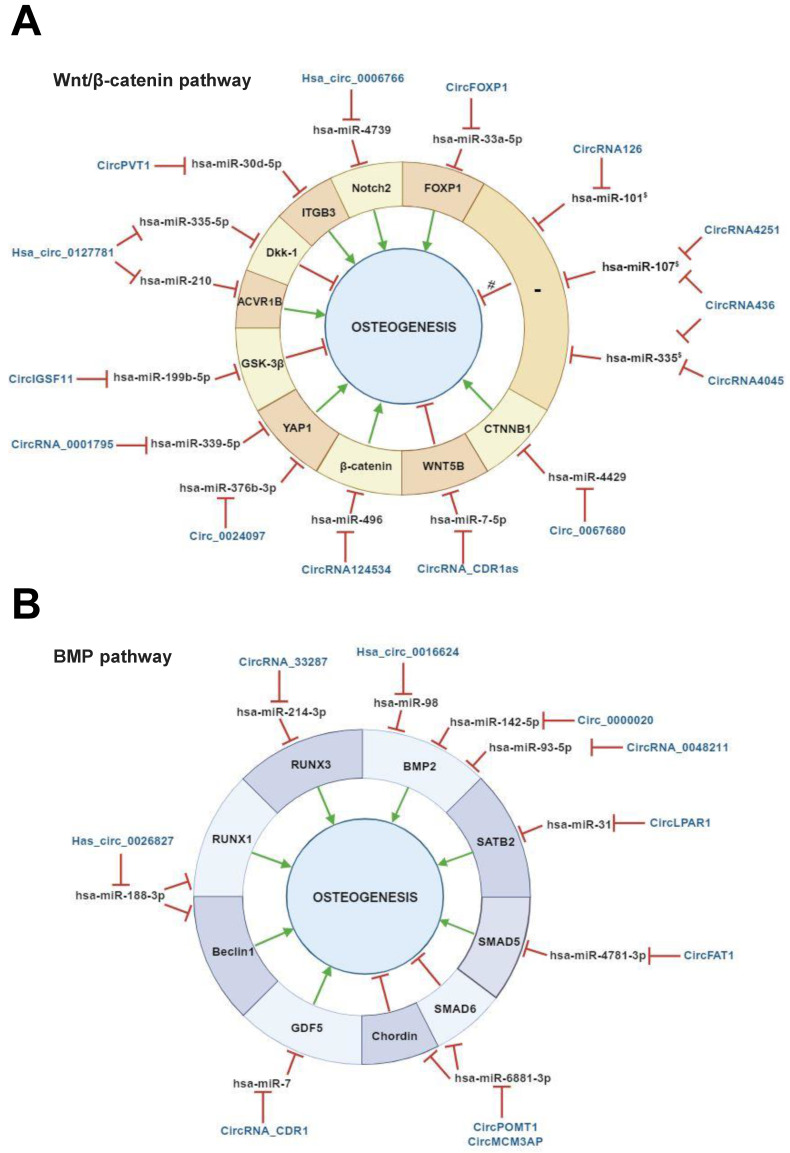
** Regulatory mechanisms of circular RNAs (circRNAs) during Wnt/β-catenin and bone morphogenetic protein (BMP)-mediated human mesenchymal stem cell (MSC) osteogenic differentiation.** CircRNAs are able to positively and/or negatively regulate MSCs osteogenesis by molecularly sponging numerous miRNAs, inhibiting, in turn, the miRNA-mediated regulatory activity on downstream osteogenic target genes. This regulatory activity can occur on different target genes implicated in **(A)** Wnt/β-catenin pathway, **(B)** bone morphogenetic protein (BMP) signaling pathway. § miRNAs previously reported as implicated in Wnt/β-catenin pathway (Zhang et al., 2017). # predicted mechanism. This figure was made by using the BioRender online tool (www.biorender.com).

The regulatory activities of circRNAs on miRNAs targeting Wnt/β‐catenin-associated genes have been documented in several studies carried out with bone marrow MSCs (BMSCs) 70,71 (Table 1, Figure 3, A). Microarray analysis performed to determine the circRNAome during BMSC osteoblast differentiation has indicated the simultaneous expression of circIGSF11 and downexpression of hsa-miR-199b-5p. Functional experiments have indicated that circIGSF11 silencing can promote osteoblast differentiation and increase miR-199b-5p expression 72. This miRNA has been reported to favor osteoblast differentiation by targeting GSK-3β/β-catenin genes 73. These data underline the potential of circIGSF11 in inhibiting osteogenesis. The same study also identified hsa_circ_0127781 as an osteogenic differentiation regulator in BMSCs. However, the role of this circRNA in osteogenesis is unclear given its inhibitory activity on both hsa-miR‐335-5p and hsa-miR‐210 72. While the first miRNA is an osteogenic inductor as a consequence of its inhibitory activity on the Wnt/β-catenin negative regulator Dkk‐1 74, the second is known to target the pro-osteoblast differentiation factor Activin A Receptor Type 1B (ACVR1B) 75. Understanding the role of hsa_circ_0127781 in human osteogenesis thus requires further research.

Several studies on BMSCs have reported that circ_0024097, circRNA_0001795, circPVT1, hsa_circ_0006766 and circ_0067680 can positively regulate the Wnt/β-catenin pathway. The first study identified circ_0024097 as an osteogenic inductor and osteoporosis inhibitor as being reported to (i) derive from yes-associated protein 1 (YAP1) gene, which interacts with β-catenin, (ii) molecularly sponge hsa-miR-376b-3p, which targets YAP1 76. The regulatory effect of circ_0024097 on hsa-miR-376b-3p can lead to the up-regulation of YAP1 expression and consequent Wnt/β-catenin signaling activation. Notably, a study aimed at evaluating the circRNA-mediated pathogenesis of osteoporosis documented that YAP1 is a downstream gene under a circRNA/miRNA axis regulation. Indeed, circRNA_0001795 has been reported to negatively regulate hsa-miRNA-339-5p in BMSCs isolated from osteoporosis, in order to prevent disease progression *via* YAP1 positive regulation 77. Similarly, circPVT1 has been reported to prompt BMSC osteogenesis and regulate osteoporosis. In BMSCs isolated from osteoporosis patients, circPVT1 expression was negatively and positively correlated with miR-30d-5p and its target Integrin beta-3 (ITGB3), respectively. ITGB3 is a component in the integrin family and a downstream gene of Homeobox D3 (HOXD3) which activates the β3 integrin-mediated WNT/β-catenin signalling. Mechanically, circPVT1 served as a molecular sponge of miR-30d-5p to increase ITGB3 expression 78. During BMSC osteogenic differentiation, hsa_circ_0006766 upregulation alongside hsa-miR-4739 downregulation has been reported 79. NOTCH2, an important gene involved in osteogenic differentiation is a hsa-miR-4739 target. These data suggest the implication of hsa_circ_0006766/miR-4739/Notch2 axis in promoting BMSC osteogenic differentiation 79. Additional data from the same study also indicated that hsa_circ_0006766 may present clinical potential as affecting the development of osteoporosis. Concerning circ_0067680, its regulatory mechanism in osteogenesis has been demonstrated in a BMSC model in which it acted as competitive endogenous RNA by sequestering has-miR-4429 to regulate the expression of Catenin Beta 1 (CTNNB1), activating, in turn, the Wnt/β-catenin pathway 80. These findings indicate that the abovementioned circRNAs can induce human osteogenic differentiation in BMSCs, and thus prompt bone formation 76,79-81.

The opposite effect for circRNAs on BMSC osteogenic differentiation has been described. Chen et al reported circRNA_CDR1as upregulation in BMSCs isolated from steroid-induced osteonecrosis of the femoral head (SONFH), which is a common debilitating orthopedic disease. Bioinformatic analyses indicated that circRNA_CDR1as could play a role in adipogenic/osteogenic differentiation-associated disorders of SONFH-BMSCs by molecularly sponging hsa-miR-7-5p, inducing, in turn, miRNA target WNT5B expression. With this regulatory mechanism, circRNA_CDR1as can counteract osteogenesis, while increasing adipogenesis in BMSCs 82.

Although no information is available on the role of circRNA_0001052 in osteogenesis, a recent study has underlined its essential function as a BMSC proliferative inductor. Liu and colleagues observed that the expression of this circRNA can decrease following Low-level laser irradiation (LLLI), a process which triggers BMSC proliferation. The same study also indicated that circRNA_0001052 can act as an hsa-miR-124-3p sponge, thus reducing BMSC proliferative potential through the Wnt/β-catenin pathway 65. Given the regulatory role of circRNA_0001052 on the Wnt/β-catenin pathway, a possible implication for this circRNA in human osteogenesis cannot be excluded, and further studies are needed to shed light on its function.

A high throughput study underlined the potential of circRNAs in regulating osteogenesis through the Wnt/β-catenin pathway in periodontal ligament stem cells (PDLSCs). In particular, circRNA126, circRNA4045, and circRNA4251 have been reported to play a role in mechanical force-induced PDLSC osteogenesis, while hsa-miR-101, hsa-miR-335, and hsa-miR-107 were computationally identified as their targets, respectively. Hsa-miR-107 and hsa-miR-335 are known to regulate MSC osteogenesis by targeting the Wnt/β-catenin pathway, while hsa-miR-107 is likely to inhibit osteosarcoma occurrence/development through Dkk‐1 downregulation 83. Additional findings have also identified circRNA436 as a regulator of PDLSC osteogenesis under mechanical force stimuli, with computationally predicted negative regulation on both hsa-miR-107 and hsa-miR-335 84. These predicted circRNA/miRNA interactions require experimental validation.

Additional studies have been carried out in human adipose stem cells (ASCs) and dental pulp stem cells (DPSCs). The pro-osteogenic activity of circFOXP1 was evaluated in a study where both *in vitro* and *in vivo* data demonstrated that this circRNA functioned as a miRNA sponge of miR-33a-5p to increase Forkhead box protein P1 (FOXP1) expression 85. The latter is known to positively regulate adipocyte and osteoblast differentiation 85. The second study reported the identification of circRNA124534 as a pro-osteogenic circRNA as a consequence of its inhibitory activity on hsa-miR-496 which target the β-catenin pathway 46. Both circRNAs can be considered as novel osteogenic inductors in humans.

To summarize, the aforementioned studies conducted mainly *in vitro* with BMSCs, PDLSCs and, to a lesser extent, ASCs and DPSCs underline the important activity of circRNAs in regulating human osteogenic differentiation through the Wnt/β-catenin pathway (Table 1, Figure 3, A).

### 4.2 Bone morphogenetic proteins (BMP) signaling pathway

Bone morphogenetic proteins (BMPs) are multifunctional transcription factors/cytokines which belong to the transforming growth factor-β family (TGF-β) (Figure 2). In humans, BMPs primarily drive osteogenesis, bone formation and chondrogenesis 86. Among the over 20 BMPs which have been identified, BMP2/4-7/9 are specifically implicated in osteogenesis. BMP signalling provides two different pathways, namely Smad-dependent and -independent pathways. In the first pathway, ligand-induced activation of Bone morphogenetic protein receptor type I-II leads to the Smad1/5/8 phosphorylation. Phosphorylated Smad1/5/8 can form a complex with Smad4, translocate into the nucleus, and act as transcriptional regulators of osteogenic downstream genes such as RUNX1/3 and Osterix 16. Several pathways are involved in the Smad-independent pathway, such as MAPK pathway, which activates ERK, JNK, and p38 87. The activation of both Smad pathways convergently promotes human osteogenesis and bone formation.

Studies with MSCs *in vitro* models showed the regulative role of circRNAs on several BMP pathway-associated genes (Table 1, Figure 3, B) 30,59,88,89. Hsa_circ_0016624, circ_0000020 and circRNA_0048211 can positively modulate BMSC osteogenesis, by acting as molecular sponges of hsa-miR-98, hsa-miR-142-5p, and hsa-miR-93-5p, respectively. These miRNAs specifically target BMP2. Hence, these circRNAs show pro-osteogenic activity in BMSCs by expressing BMP2. As a support, circRNA_0016624 and circRNA_0048211 are known to prevent osteoporosis 90,91. Another study reported the evaluation of the circRNAome during osteogenic differentiation of BMP2 induced-maxillary sinus membrane stem cells (MSMSCs). The main findings on circRNA_33287 indicated its (i) up-regulation throughout MSMSC differentiation, (ii) molecular sponge activity on miR-214-3p, which is known to regulate Runt-related transcription factor 3 (RUNX3) expression. A pro-osteogenic effect and ectopic bone formation stimulation were afterward confirmed for this circRNA *in vivo* 35.

Two PDLSC-based study models demonstrated the regulative activity of circRNAs on BMP-driven osteogenic differentiation. In particular, circFAT1 and circRNA_CDR1 were identified as players in PDLSC osteogenesis. CircFAT1 presented pro-osteogenic activity in PDLSCs by acting as a molecular sponge of hsa-miR-4781-3p which is known to target SMAD5 92. CircRNA_CDR1 demonstrated inhibitory activity on hsa-miR-7, triggering, in turn, the (i) upregulation of the BMP pathway-associated gene Growth/differentiation factor 5 (GDF5), (ii) Smad1/5/8 phosphorylation. This mechanism has been reported to ultimately promote PDLSC osteogenesis 93. These studies provide a novel understanding of the mechanisms of circRNA-driven osteogenesis and PDLSC-mediated periodontal bone regeneration.

Studies conducted with DPSCs identified hsa_circ_0026827 and circLPAR1 as osteogenic regulators *via* BMP pathway modulation. Hsa_circ_0026827 can promote osteoblast differentiation *via* Beclin1 and RUNX1 signaling by molecularly sponging their inhibitory miRNA hsa-miR-188-3p 94. CircLPAR1 plays a role in osteogenesis by eliminating the inhibitory effect of hsa-miR-31 on its target Special AT-rich sequence-binding protein 2 (SATB2). This gene is a potent pro-osteoblastogenic transcription factor that promotes bone generation by driving the upregulation of pro-osteogenic downstream genes 95.

With the aim of evaluating the regulatory potential of circRNAs during ASCs osteogenic differentiation, Huang et al identified circPOMT1 and circMCM3AP as negative regulators of this biological process. Both circRNAs demonstrated negative regulation of hsa-miR-6881-3p, which is known to promote osteogenesis by targeting two critical BMP pathway inhibitors named Smad6 and Chordin. Given their anti-osteogenic activity, circPOMT1 and circMCM3AP can be considered as potential candidate targets for the bone defect repair 96.

### 4.3 Wnt/β-catenin and Bone morphogenetic proteins (BMP) signalling pathways

**Figure 4:**
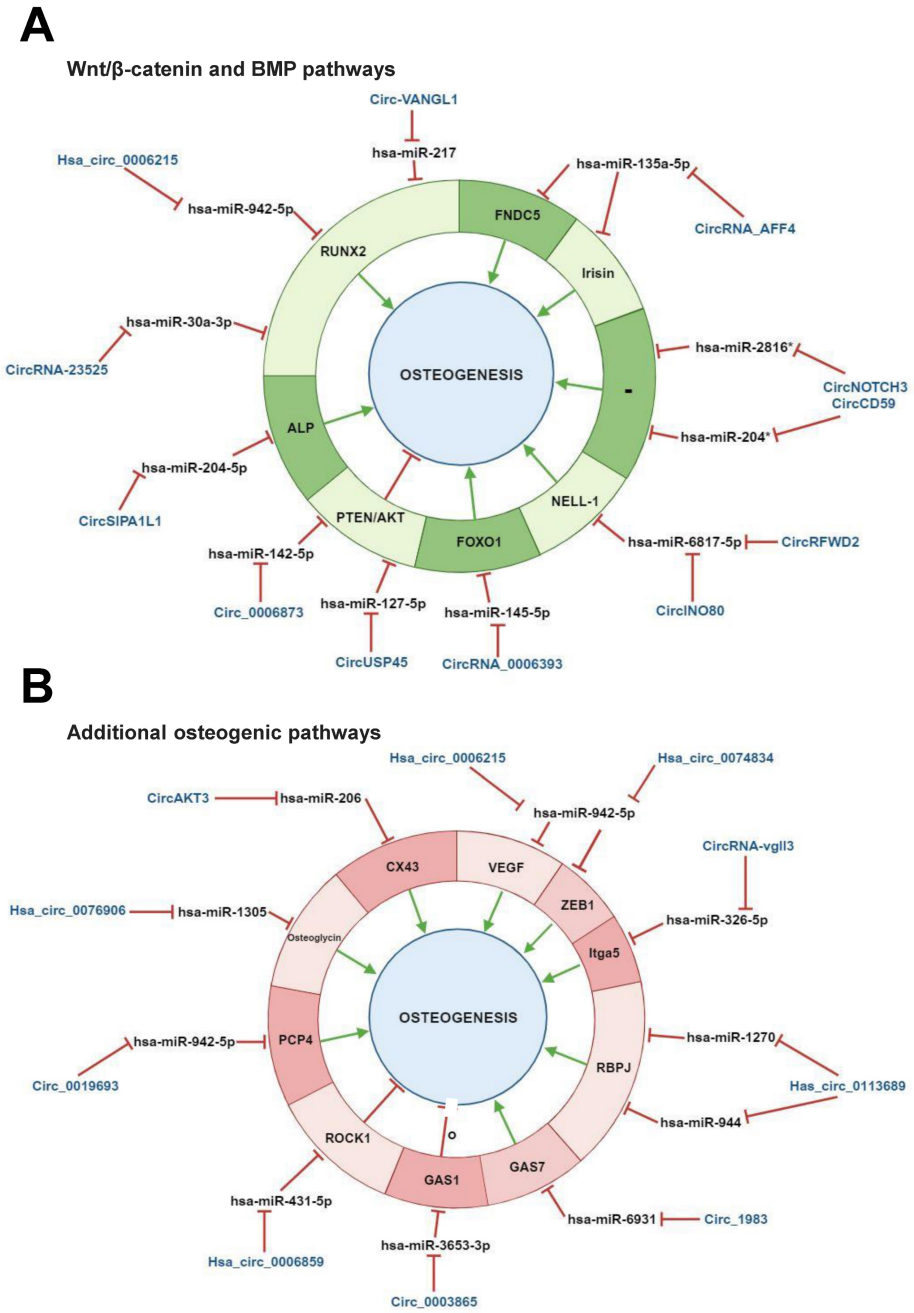
** Regulatory mechanisms of circular RNAs (circRNAs) during human mesenchymal stem cell (MSC) osteogenic differentiation mediated by both Wnt/β-catenin and bone morphogenetic protein (BMP) signaling pathways and by additional pathways involved in human osteogenesis.** CircRNAs are able to positively and/or negatively regulate MSCs osteogenesis by molecular sponging numerous miRNAs, inhibiting, in turn, the miRNA-mediated regulatory activity on downstream osteogenic target genes. This regulatory activity can occur on different target genes implicated in **(A)** both Wnt/β-catenin and bone morphogenetic protein (BMP) signaling pathways, **(B)** additional pathways involved in osteogenesis. *Predicted interaction. These miRNAs have been reported to be implicated in Both Wnt/β-catenin and BMP pathways (Huang et al., 2010, Diomede et al., 2016). This figure was made by using the BioRender online tool (www.biorender.com).

Wnt/β-catenin and BMP signalling pathways are known to cooperatively regulate human osteogenesis. These pathways particularly overlap in the activation of the osteogenesis master regulator RUNX2 1. RUNX2 expression induces the activation of several downstream osteogenic proteins, including Osteocalcin, Osteopontin and alkaline phosphatase 97. CircRNAs have been reported to regulate human osteogenesis by modulating the expression of converging factors from both pathways (Table 1, Figure 4, A).

Several studies carried out mainly *in vitro* with BMSCs isolated from bone diseases patients reported RUNX2 regulation through various circRNA/miRNA axes. A recent study has demonstrated an important function for hsa_circ_0006215 in BMSC osteogenesis by regulating cell aging in senile osteoporosis patients and promoting bone defects repair. Hsa_circ_0006215 has been related to RUNX2-driven osteogenesis as it can molecularly sponge hsa-miR-942-5p, which targets RUNX2 49. Dysregulated RUNX2 expression is frequently associated with the onset of osteoarticular diseases. It has been closely linked to bone formation and hypertrophic chondrocyte differentiation, which is a physiological stage in endochondral ossification starting from MSC condensation 98. Consistently, abnormal down‐regulated RUNX2 expression could lead to bone formation/mass inhibition/decrease 99,100. Yin et al. demonstrated that circRUNX2 can target hsa-miR‐203, in order to prevent osteoporosis *via* RUNX2 positive regulation 101. A similar effect has been reported for circ-VANGL1, whose decreased expression in BMSCs isolated from osteoporosis patients was related to the negative modulation of RUNX2 and osteoporosis promotion. Low circ-VANGL1 levels also prevented the inhibitory activity of this circRNA on hsa-miRNA-217, which is known to target RUNX2. This impaired mechanism negatively modulates several RUNX2 downstream pro-osteogenic proteins such as bone sialoprotein, osteocalcin, and osteopontin as well as enhance disease progression 102,103. These overarching findings may allow circRNA/miRNA axes to be exploited as novel therapeutic targets for osteoporosis treatment 101,103. RUNX2 modulation has also been demonstrated in adipose-derived mesenchymal stem cells (ADSCs). In this study, hsa_circRNA-23525 was identified as an osteogenic regulator by positively regulating RUNX2 *via* molecularly sponging its inhibitory miRNA miR-30a-3p. This molecular interaction prompted the ADSC osteoblastic differentiation 104.

Additional studies on BMSCs identified circ_AFF4, hsa_circ_0006393 and circUSP45 as osteogenic regulators. Circ_AFF4 sponges hsa-miR-135a-5p to prompt the expression of the miRNA target Fibronectin type III domain-containing protein 5 (FNDC5)/irisin, which belongs to the Smad1/5 pathway 105.

Irsin is a myokine and a proteolytic cleavage product of FNDC5, which is involved in osteogenesis as an MAPK pathway activator 106,107. Hsa_circ_0006393 can lead to the overexpression of the pro-osteogenic gene Forkhead box protein O1 (FOXO1) by sponging its miRNA inhibitor hsa-miR-145-5p. This mechanism has been related to osteoblast proliferation and bone mass increase 108. Both circ_AFF4 and hsa_circ_0006393 therefore exhibited a pro-osteogenic potential in BMSCs. A negative effect on osteogenesis has instead been demonstrated for circUSP45. This circRNA has been reported as being overexpressed during glucocorticoid-induced osteonecrosis of PBMCs isolated from femoral head (GIONFH) patients. Moreover, circUSP45 knockdown can prevent hsa-miR-127-5p sponging, thus allowing the negative regulation of this miRNA on its target Phosphatase and tensin homolog (PTEN) 109, promoting, in turn, BMSC proliferation and osteogenesis 110. PTEN is known to play a role as a direct inhibitor of AKT and is involved in blocking cell growth and in the induction of apoptosis 111. An additional study aimed at evaluating the role of circ_0006873 in osteoporosis, reported that PTEN/AKT control also encompasses circ_0006873/miR-142-5p axis regulation. Circ_0006873 can molecularly sponge miR-142-5p thereby enhancing PTEN expression to suppress osteoblastic differentiation and favor osteoporosis 112. The aforementioned study models highlighted the potential of circRNAs in control human osteogenesis by regulating predominantly RUNX2 and, to a lesser extent, other Wnt/β-catenin and BMP signaling pathway converging factors.

Additional studies on ASCs and stem cells from apical papilla (SCAPs) identified several circRNAs as Wnt/β-catenin and BMP signalling pathway regulators. CircRFWD2 and circINO80 have been identified as pro-osteogenic circRNAs in an ASC model. In particular, the upregulation of both molecules has been reported during Protein kinase C-binding protein (NELL‐1)‐induced osteogenesis in human ASCs. Intriguingly, both circRNAs can convergently sponge hsa-miR‐6817‐5p, which is a negative modulator of NELL-1, and enhance, in turn, NELL-1-mediated osteogenesis 113. Alkaline phosphatase is an important MSC differentiation regulator and plays an inhibitory role in bone aging 114. Hsa-miR-204-5p has recently been identified as its specific targeting miRNA. This miRNA, an osteoblast aging regulator, is able to lead to Wnt/beta-catenin pathway suppression and RUNX2 downregulation 115,116. During SCAPs osteogenic differentiation, hsa-miR-204-5p downexpression has been related to the overexpression of circSIPA1L1. In particular, circSIPA1L1 can inhibit the negative modulation of hsa-miR-204-5p on alkaline phosphatase, ultimately leading to osteogenic differentiation 117.

Lastly, an early study carried out to determine the circRNA landscape during PDLSC osteogenesis, identified circNOTCH3 and circCD59 as osteogenic players. Despite a large catalogue of circRNAs and enriched functions being identified, *in silico* findings identified circNOTCH3 and circCD59 as hub circRNAs involved in osteogenesis by molecularly sponging their hsa-miR-204 and hsa-miR-2816 targets 118. Previous studies reported that hsa-miR-204 can inhibit MSC osteogenesis and induce adipogenesis by targeting RUNX2, while hsa-miR-2816 can enhance osteoblast differentiation through RUNX2 overexpression 119,120. The potential implication of circNOTCH3 and circCD59 in osteogenesis requires further experimental, functional validation.

To summarize, the control of human osteogenic differentiation has frequently been reported to encompass the regulation of interconnected Wnt/β-catenin and BMP signalling pathways through circRNA/miRNA axis activities (Table 1, Figure 4, A).

### 4.4 Additional pathways involved in osteogenic differentiation

Studies conducted mainly *in vitro* with BMSCs showed that circRNAs can regulate pathways associated with canonical human osteogenic differentiation pathways (Table 1, Figure 4, B). Besides Wnt/β-catenin and BMP pathways, additional pathways, such as the vascular endothelial growth factor (VEGF) pathway, play a role in osteogenesis. Given the high vascularization of bones, angiogenesis is highly related to osteogenesis and these two processes can be mutually influenced by common players. VEGF plays a role in this context as a well-known master regulator of angiogenesis, while a function for this gene as a skeletal development and bone repair has also been documented 121. Hsa_circ_0006215, hsa_circ_0074834 and circ_0019693 have been identified as positive regulators of BMSC osteogenesis-coupled angiogenesis. Intriguingly, distinct studies have indicated that these circRNAs can simultaneously counteract the inhibitory ability of hsa-miRNA-942-5p on its targets VEGF and RUNX2 as well as ZEB1 and PCP4, consequently favoring the osteogenesis-angiogenesis coupling process 49,81. Both ZEB1 and PCP4 play a role in osteogenesis, while the latter has also been associated with calcium deposition during BMSC osteogenesis 9,122. Moreover, since both hsa_circ_0006215 and circ_0019693 have been identified at low levels in BMSCs and sera, respectively, from osteoporosis patients, the putative function of these circRNAs as candidate therapeutic targets cannot be excluded 9,49. Furthermore, hsa_circ_0006215 has been reported to enhance bone formation *in vivo* in a cortical bone defect model 49. These studies cumulatively demonstrate that circRNAs can promote the osteogenesis-angiogenesis coupling process through different regulatory mechanisms, including the negative modulation of the inhibitory activity of the same miRNA on multiple target genes.

As an alternative mechanism, the same circRNA can negatively regulate multiple miRNAs involved in osteogenesis-associated pathways. For instance, has_circ_0113689 (or circ-DAB1) can molecularly sponge two distinct miRNAs, namely hsa-miR-1270 and hsa-miR-944. BMSC osteogenesis is improved given the repression of the inhibitory activity of these two miRNAs on their target Recombination signal Binding Protein for immunoglobulin kappa J (RBPJ), which is a NOTCH pathway-related transcription factor. It should be recalled that NOTCH pathway facilitates human osteogenesis 19.

A recent study has reported that circRNAs can control the balance between BMSC osteogenesis and adipogenesis, by regulating factors from osteogenesis-associated pathways. A microarray analysis identified has_circ_0006859 as one of the most upregulated circRNAs in sera from postmenopausal osteoporotic patients. Functional data indicated that has_circ_0006859 can molecularly sponge hsa-miR-431-5p, counteracting, in turn, the inhibitory ability of hsa-miRNA-942-5p on its target ROCK1, which is known to regulate cell motility. This circRNA/miRNA/mRNA crosstalk was able to suppress BMSC osteoblastic differentiation and even promote adipogenesis, as an increase in lipid droplet formation was observed, too 123.

Additional studies with BMSCs showed the regulative role of circ_0003865, circ_1983, and circ_0076906 on different osteogenesis-associated pathways 99. With the aim of evaluating the implications of circRNAs on the effect of melatonin treatment on BMSC osteogenic differentiation and osteoporosis, Wang and colleagues identified circ_0003865. Melatonin is known to stimulate osteoblast proliferation/differentiation by promoting bone formation and alleviating bone destruction in osteoporosis mice 124. Circ_0003865 expression was reported as decreased in melatonin treated in BMSCs. Mechanistically, circ_0003865 hampered osteogenesis by molecularly sponging hsa-miR-3653-3p, ultimately favoring the expression of the cell cycle regulator GAS1 125. Concerning circ_1983, a recent study aimed at evaluating the molecular effect of dicalcium silicate microparticle (C_2_S)-based biomaterials in BMSCs, described the upregulation of this circRNA in treated cells 126. The inhibitory activity of circ_1983 has also been demonstrated functionally on hsa-miR-6931. The molecular sponging of hsa-miR-6931 by circ_1983 induced the enhanced expression of the miRNA target Growth arrest-specific protein 7 (GAS7), thus favoring C_2_S-treated BMSC osteogenic differentiation. Lastly, hsa_circ_0076906 has recently been found to sponge hsa-miR-1305 and consequently induce the positive regulation of its target gene, osteoglycin, to favor MSC osteogenesis and alleviate osteoporosis 127. As such, hsa_circ_0076906 may present clinical utility for osteoporosis. These studies cumulatively indicate that osteogenic differentiation of MSCs/BMSCs encompasses additional osteogenesis-associated pathways whose players are under circRNA/miRNA axis regulation.

A few studies conducted in DPSCs and ADSCs explored the role of circRNAs in regulating genes involved in additional osteogenesis-associated pathways 128. Zhang et al. reported circAKT3 as a positive regulator of osteogenic differentiation by counteracting the inhibitory activity of hsa-miR-206 on its target Connexin 43 (CX43) in DPSCs. In particular, the inhibition of hsa-miR-206 induced CX43 expression, which is a gap junction protein and an osteogenesis regulator. The same group also demonstrated that circAKT3 knockdown *in vivo* can block the (i) formation of mineralized nodules, (ii) expression of pro-osteogenic proteins 129. One recent circRNA-based study conducted on ADSCs has documented that circRNA-vgll3 plays a role in improving osteogenesis through the hsa-miR-326-5p/integrin α5 (Itga5) axis. In particular, circRNA-vgll3 can molecularly sponge hsa-miR-326-5p, thus preventing the inhibitory effect of this miRNA on Itga5 and consequently promoting the ADSC osteogenic differentiation 130.

To summarize, the aforementioned studies highlighted that human osteogenic differentiation control encompasses the regulation of additional osteogenesis-associated pathways through circRNA/miRNA axis activities (Table 1, Figure 4, B).

## 5. Future perspectives

The identification of circRNAs as key osteogenic regulators is increasing the knowledge on the molecular processes at the basis of bone growth in humans 131-133. The development of novel bioinformatic tools and experimental methods 134, such as functional approaches and improved cell culture models 135-138, are rapidly expanding our understanding of the roles played by circRNAs in osteogenesis 139,140. From this perspective, human stem cells from various sources display biological properties that are useful for studying circRNAs 141. hMSCs can be collected from different anatomical areas, such as bone marrow, adipose tissue, periodontal ligament, dental pulp, and apical papilla of the tooth, and be valuable biological sources for studying circRNA functions and mechanisms 142.

The large fraction of studies described herein have been conducted using *in vitro* settings mainly based on human BMSC models. Data on circRNAs obtained in human PDLSC, ASC and SCAP models have also been reported, although in a limited number of studies (Table 1). Human bone regeneration is a complex and well-orchestrated process which involves a plethora of molecular factors/pathways and cellular/physiological processes. For a more comprehensive understanding, the study of circRNAs should be enlarged to stem cells isolated from multiple human anatomical sources. Moreover, validation *in vivo* with animal models of the data being obtained *in vitro* has been reported in a small number of studies 35,49,81,85,124,129. Evaluating the molecular activities of circRNAs during human osteogenesis and bone regeneration in a complex *in vivo* model is far more accurate and informative than *in vitro*. We thus encourage further research which should focus on the development of animal models-based experimental designs.

Overall, the studies mentioned in this review have demonstrated the pivotal role of circRNAs in modulating human osteogenic differentiation through interaction with miRNAs. CircRNA/miRNA interplay has been demonstrated to induce predominantly a positive effect on osteogenesis which was supported by the positive regulation of numerous downstream factors involved in Wnt/β-catenin, BMP and additional osteogenesis-associated pathways. It should be noted, however, that a modest number of circRNAs has also been reported to negatively regulate human osteogenesis (Table 1).

Although the regulatory mechanisms of circRNAs in human osteogenesis have been explored to some extent, limited information is currently available on the role of circRNAs in bone homoeostasis 143,144, which provides the delicate balance between osteogenesis and osteoclastogenesis. Indeed, to the best of our knowledge, a few circRNAs including circRNA-28313, circRNA_009934, circ_0008542 and circ_0021739 have been reported as human osteoclastogenesis players 145-148. Similarly, the knowledge behind the implication of dysregulated circRNAs in the initiation and progression of human bone diseases 149, such as osteosarcoma, osteoporosis and osteonecrosis is still in its early phases 150-153. Given the increasing potential clinical application of circRNAs in cancer as biomarkers and/or therapeutic targets, it might be a fruitful work to study the oncogenic role of these molecules in osteosarcoma. However, the research in this field is still relatively limited, compared to other tumors 150. Several circRNAs reported in this review such as hsa_circ_0006766, circRNA_0001795, circPVT1, circRNA_0016624, circRNA_0048211, hsa_circ_0006215, circ_0006873, circRUNX2, hsa_circ_0006215, circ_0019693, hsa_circ_0006859, circ_0003865, hsa_circ_0076906 have been reported as involved in osteoporosis occurrence 9,49,77-79,90,112,123,125,127,154,155. However, the implication of the remaining circRNAs described herein in the initiation/development of both osteoporosis and osteonecrosis is still unclear. Additional research is thus required to translate the relevance of regulatory mechanisms of circRNAs into the clinic. It is imperative to further study the implication of circRNAs not only in the context of human bone homeostasis but also by analyzing the dysregulated mechanisms underlying human bone diseases.

It is worth nothing that circRNA activity as competitive endogenous RNAs for suppressing the miRNA inhibitory effect on downstream target genes is the only molecular mechanism documented so far in human osteogenesis. However, researchers should center around additional circRNA-mediated mechanisms such as the (i) regulation of the circRNA parental gene in terms of epigenetic control, splicing, transcription, or translation, (ii) circRNA-protein binding potential, (iii) circRNA protein and/or peptide translation potential (Figure 1) 156-160. Several circRNAs which mediate these mechanisms might be of interest in osteogenesis. For instance, the circRNA circ-DONSON has been reported to recruit the chromatin remodelling complex NURF to initiate the expression of SOX4, which is a transcription factor being implicated in osteogenesis, bone formation and even osteoporosis 161. Moreover, an additional circRNA, i.e., circ-Foxo3, has been reported to interact with p21 and CDK2 in order to regulate cell cycle progression and survival of non-cancer cells 162. Notably, Foxo3 gene is known to regulate the osteogenic differentiation of mesenchymal stem cells 163. It is therefore clear that the regulatory mechanisms whereby circRNAs regulate osteogenesis should be more deeply investigated and enriched. Understanding these mechanisms will enhance our comprehension of the osteogenic differentiation process resulting in the improvement of medical treatments for bone diseases. In the future, a better understanding of the mechanisms of action of circRNAs and their relationship with Wnt/β-catenin and BMPs signaling pathways will allow to determine potential therapeutic targets for the treatment of human bone disorders.

## 6. Conclusions

In conclusion, this review summarizes the most recently published findings on the regulative mechanisms of circRNAs during human osteogenic differentiation 164,165. The majority of the studies have been carried out in BMSCs, which is the reason why further studies are needed on MSCs isolated from other human anatomic districts. Furthermore, some *in vivo* studies are beginning to validate the biological role of the molecular functions of circRNAs, but more research with animal models is required. We can definitely predict that circRNAs will have a greater influence on human osteogenic differentiation, due to their multiple novel molecular activities. We believe that the recent findings on various circRNA/miRNA/mRNA interplays described in this review will lead to a better understanding of the molecular mechanisms of human bone remodeling and related bone disorders. Recent discoveries on circRNAs will encourage the development of novel preclinical and clinical studies, eventually resulting in new therapeutic approaches in the treatment of human bone disorders.

## Figures and Tables

**Table 1:**
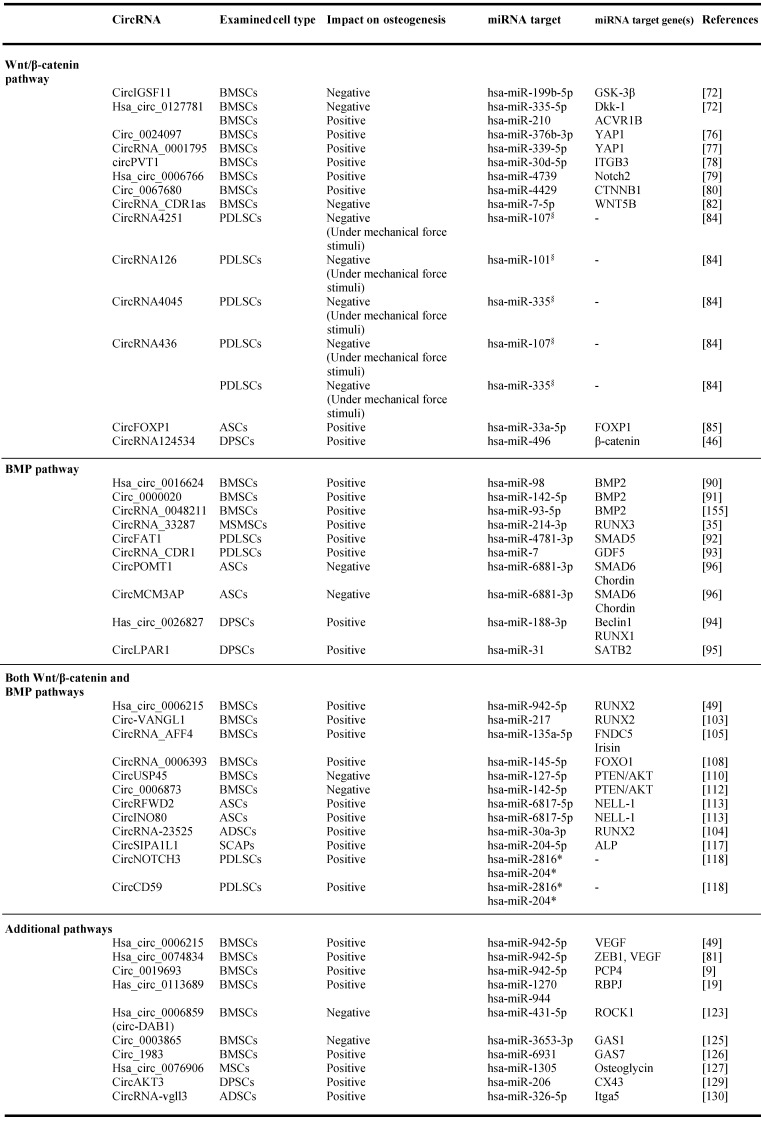
Relationship between circular RNAs, microRNAs and mRNAs during osteogenesis.

^§^ miRNAs implicated in Wnt/β-catenin pathway (Zhang et al., 2017). *Predicted interaction of circRNA implicated in Both Wnt/β-catenin and BMP pathways (Huang et al., 2010, Diomede et al., 2016). Abbreviations: Mesenchymal stem cells (MSCs), bone marrow mesenchymal stem cells (BMSCs), periodontal ligament stem cells (PDLSCs), human adipose stem cells (hASCs), dental pulp stem cells (DPSCs), maxillary sinus membrane stem cells (MSMSCs) adipose derived mesenchymal stem cells (ADSCs), stem cells from apical papilla (SCAPs).
